# Nonsyndromic cleft lip and/or palate: A multicenter study of the dental anomalies involved

**DOI:** 10.4317/jced.54926

**Published:** 2018-08-01

**Authors:** Carlos Menezes, José-Alcides de Arruda, Leni-Verônica Silva, João-Luiz Monteiro, Pérola Caribé, Pamella Álvares, Maria-Cristina Almeida, José-Carlos Coelli, Fernanda Goldemberg, Marcia Silveira, Ana-Paula Sobral, Daniela-Franco Bueno

**Affiliations:** 1 Institute of Teaching and Research of Hospital Sírio Libanês, São Paulo, SP, Brazil; 2 Department of Oral Surgery and Pathology, School of Dentistry, Universidade Federal de Minas Gerais, Belo Horizonte, MG, Brazil; 3 Department of Oral and Maxillofacial Surgery, School of Dentistry, Universidade de Pernambuco, Camaragibe, PE, Brazil; 4 Department of Oral and Maxillofacial Pathology, School of Dentistry, Universidade de Pernambuco, Camaragibe, PE, Brazil; 5 Department of Orthodontics, Hospital Municipal Infantil Menino Jesus, São Paulo, SP, Brazil; 6 Department of Orthodontics, Centrinho Prefeito Luiz Gomes, Joinville, SC, Brazil

## Abstract

**Background:**

Nonsyndromic cleft lip and/or palate (NSCL/P) is the most common craniofacial malformation. Due to the anatomical defect present in the alveolar process, these patients tend to exhibit more dental anomalies. The aim of this study was to identify the prevalence of dental anomalies in patients with NSCL/P by obtaining orthodontic documentation from Brazilian Centers for cleft lip and palate treatment.

**Material and Methods:**

A retrospective analysis (2000-2014) was conducted on orthodontic archives, radiographs and medical records of NSCL/P of 524 patients under orthodontic treatment. Panoramic radiographs and intra-oral photographs were examined to identify these anomalies. Categorical variables were expressed in terms of frequencies and percentages and analyzed using the Chi-Square test. The level of significance was set at *p*≤0.05 in all analyses.

**Results:**

Approximately 83.3% of the individuals had at least one dental anomaly. Tooth agenesis was the most common abnormality found in those patients (87.8%) (*p*<0.001). Also, the largest number of dental anomalies was detected in the group of unilateral left clefts. The prevalence of dental anomalies in the present sample of NSCL/P patients was high and reached the highest levels in patients with alveolar bone clefts.

**Conclusions:**

This study describes the most common dental anomalies observed in patients with NSCL/P. These abnormalities can cause significant problems that may be solved or minimized by early diagnosis and treatment.

** Key words:**Cleft lip and/or palate, dental care for children, epidemiology, craniofacial abnormalities.

## Introduction

Cleft lip (CL), cleft palate (CP) and cleft lip and palate (CLP) are the most common craniofacial malformations detected at birth, representing 25% of all congenital craniofacial defects. Approximately 1 in 700 live births has a kind of cleft. A multifactorial model of genetic inheritance has been suggested for nonsyndromic cleft lip and/or palate (NSCL/P) based on the interaction of genetic and environmental factors (1,2). It is very common to observe different kinds of tooth anomalies in children with cleft lip and/or palate (CL/P), such as supernumerary teeth or missing teeth, microdontia, rotated teeth, hypoplasia, transpositions, and root deviation, usually detected on the cleft side (3-5). Tooth anomalies can be a challenge to the multidisciplinary health team treating these patients, but early detection improves treatment outcomes and orthodontic and surgical planning can be properly executed to obtain adequate aesthetic and functional results (3-9).

The development of CL/P and tooth germs involves a very close embryologic relationship in terms of timing and anatomical position (3,6,10,11). Johnson *et al.* (12) have suggested that gene alterations associated with oral clefts can also produce many disturbances in other tissues, including changes in the dental lamina signaling pathway during odontogenesis, leading to dental malformations.

Data about the prevalence of dental anomalies in Brazilians NSCL/P are scarce. Therefore, to expand our knowledge about this topic, we evaluated the samples of three referral centers located in representative regions of Brazil (Northeast, Southeast and South).

## Material and Methods

-Study design and ethical approval

Data regarding 524 patients with NSCL/P were analyzed in a retrospective analysis (2000-2014). Data were obtained from three referral centers in Brazil: Instituto Materno Infantil de Pernambuco, in Pernambuco (Northeast region), Hospital Municipal Infantil Menino Jesus, in São Paulo (Southeast region), and Centrinho Prefeito Luis Gomes, in Santa Catarina (South region). The hospitals involved in this study are reference centers for the treatment and rehabilitation of individuals with any kind of malformation, including oral clefts. However, only NSCL/P patients were included in the present investigation. The study was approved by the Ethics Committee of the Sírio Libanês Hospital (Approval No. 0020.097.000-09) and was conducted in accordance with the guidelines of the Declaration of Helsinki.

-Sample

Data regarding gender, age, type of cleft and dental anomalies were collected from a total of 524 patients with NSCL/P aged ≤18 years. The patients were divided into groups according to the Spina (13) classification of clefts: unilateral right CL/P (URCLP), unilateral left CL/P (ULCLP), bilateral CL/P (BCLP), and cleft palate only (CP). Form and number of anomalies were considered, excluding the third molar. One method for the assessment of the presence of dental anomalies was the use of panoramic and periapical radiographs, with the evaluators being blind to possible records of these anomalies in the medical records. Furthermore, intra-oral photographs were retrieved. All patients were evaluated by a geneticist in order to determine and exclude any case presenting an association between CL/P and some syndrome.

The radiographs were analyzed by four independent specialists (an oral and maxillofacial pathologist, an oral and maxillofacial radiologist and two orthodontists) with more than 20 years of experience. Radiographs with acceptable sharpness, contrast and density were considered in the analysis. Records with previous tooth extractions and incomplete CL/P description, as well as radiographs with poor image quality were excluded.

-Data analysis

Categorical variables are reported as frequencies and percentages and were analyzed by the Chi-square test and the Fisher exact test, with the level of significance set at 5% (*p*≤0.05) in all analyses. Statistical modeling and the tests were performed using Statistical Package for the Social Sciences software, version 21.0 (SPSS, Inc., Chicago, IL, USA).

## Results

Of the 524 patients studied, 283 were males (54%) and 241 were females (46%). Regarding the number of patients who were undergoing orthodontic treatment at each reference center, 231 (44% of the total sample) were treated at Instituto Materno Infantil de Pernambuco (Northeast region), 156 (29.7% of the total sample) at Hospital Municipal Infantil Menino Jesus (Southeast region), and 137 (26.1% of the total sample) at Centrinho Prefeito Luis Gomes (Southern region).

**Figure 1:**
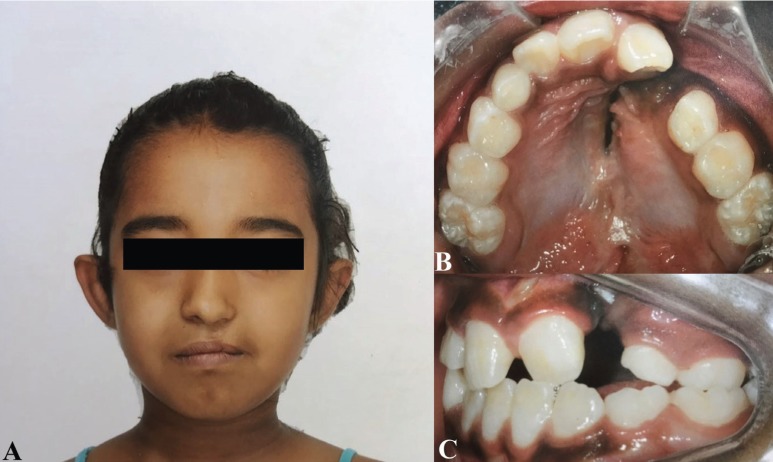
A. A 9-year old patient showing repaired unilateral left cleft lip and palate. B. Occlusal view of cleft lip and palate. C. Lateral view of cleft lip and palate showing the lateral agenesis.

According to the Spina (13) classification, 105 patients had URCLP (20%), 224 had ULCLP (42.7%) (Fig. 1), 110 had BCLP (20.9%), and 85 had only CP (16.2%) (*p*<0.001) ( Table 1).

**Table 1:**
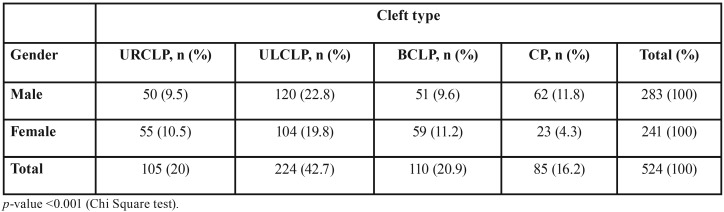
Distribution of patient according to type of cleft and gender.

**Figure 2:**
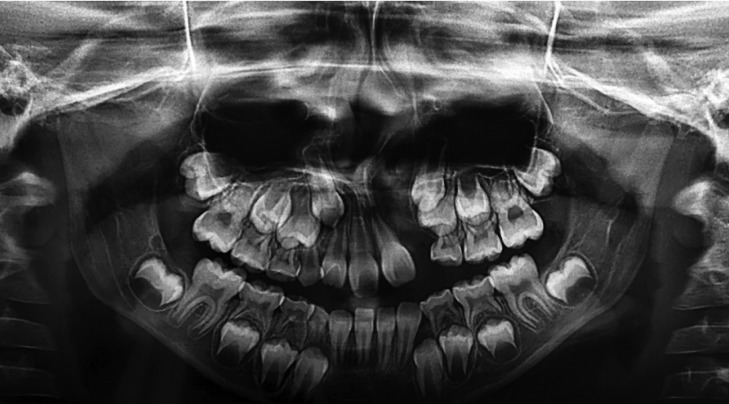
Panoramic radiograph showing agenesis of the left lateral incisor.

At least one dental anomaly was observed in 437 patients, corresponding to 83.3% of the total sample (*p*<0.001) ( Table 2). The most frequent dental anomaly was tooth agenesis (Fig. 2), which occurred in 271 out of 437 patients, representing 62% of all dental anomalies in this study. Agenesis of the upper lateral incisors on the alveolar cleft side was the anomaly most frequently observed (87% of all cases of tooth agenesis) ( Table 2).

**Table 2:**
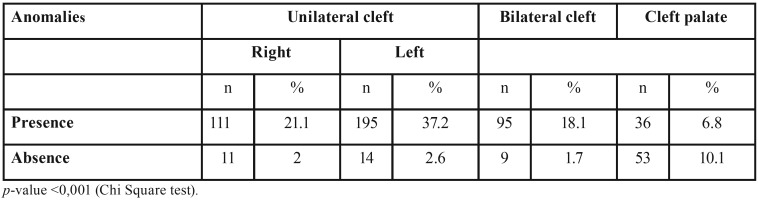
Distribution of patient according to type of cleft and presence or not of dental anomalies.

Rotated tooth was the second tooth abnormality most frequently detected in this sample (n=142, 32.4%), with the central incisor being the tooth most frequently involved. A total of 104 patients had supernumerary teeth (23.7%) and 78 patients had impacted teeth (17.8%), with the canine being the most affected tooth. The other dental anomalies observed were: microdontia (13%), ectopic tooth (6.4%), hypoplasia (3.2%), dilacerations (0.9%), and hypercementosis (0.6%) ( Table 3).

**Table 3:**
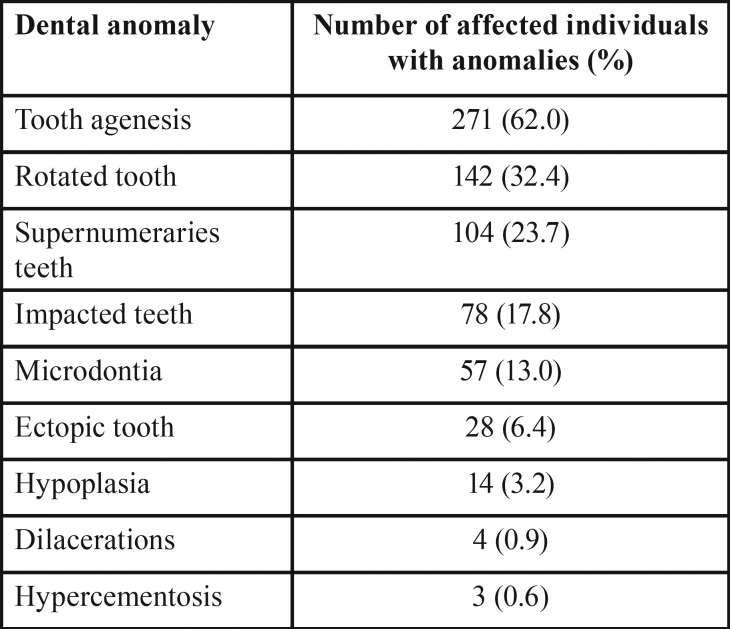
Frequency of dental anomalies in 437 patients with NSCL/P.

## Discussion

Considering the high incidence of clefts around the world, it is extremely important for the multidisciplinary health team to be familiarized with dental anomalies in order to carry out the correct orthodontic and surgical treatment for these children and adolescents. The aim of the present study was to determine the prevalence of dental anomalies in NSCL/P patients at reference centers in distinct regions of Brazil.

To the best of our knowledge, this is the first multicenter study conducted in Brazil which describes the prevalence of dental anomalies in NSCL/P patients using data obtained from patients who were undergoing orthodontic treatment at specialized centers located in three Brazilian regions (Northeast, Southeast and South). In this study we observed that at least 83.3% of our NSCL/P sample had some type of tooth alteration. A recent study which investigated a large cohort of children with NSCLP, their relatives, and controls found a significantly higher risk for dental anomalies compared to the general population. However, the findings suggested that the affected families did not have a higher genetic risk for dental anomalies than the general population and that the higher prevalence of these anomalies was mainly a consequence of various surgical interventions (14). However, this higher prevalence (3,11,15,16) of dental anomalies observed in cleft patients has been reported to be related to the existence of a common genetic link between oral clefts and dental anomalies in other studies (17). For instance, genes whose mutations are associated with tooth agenesis, such as MSX1 and PAX9, also contain single nucleotide polymorphisms as genetic risk factors for orofacial clefts (18). Other studies have shown that the severity of dental anomalies varies according to the complexity of the clefts (4,19).

The most common tooth alteration observed in our sample was tooth agenesis (62% of all dental anomalies). Tooth agenesis occurs approximately three times more frequently on the cleft than on the non-cleft side (20). The upper lateral incisor was the most common tooth missing on the alveolar cleft side, representing 87% of all cases of dental agenesis detected in our study. Other recent studies on NSCL/P conducted in Italy (21), and in Greece (22) also agree with these findings. It is important to identify this lateral incisor agenesis before performing an alveolar bone graft in CL/P patients since this anomaly affects surgical and orthodontic planning. For instance, the space may be maintained for future placement of a dental implant, or the canine may be moved closer.

Other abnormalities are microdontia, hypoplasia, impaction, rotation, peg teeth, and root deviation (23,24). Anodontia can occur by absent or incomplete development of the tooth germ, (23,24). Three hypotheses have been raised for supernumerary teeth: the first states that development starts with remnants of epithelial cells from the disintegration of the dental lamina; the second suggests that development occurs from the complete division of a dental germ, and the third and most accepted one states that hyperactivity of the dental lamina occurs, causing invagination from the oral epithelial lining (25). In addition, peg teeth are frequently found in CL/P patients. It is most common in cases where the lateral incisor is not absent but has a deformed shape (25). Hypoplasia is another dental anomaly found at higher frequency in patients with CL/P, affecting more commonly the central incisors (4,24,25).

The impaction of canines in the permanent dentition is very common in these individuals. This change in position has an important impact on the development of dentition and occlusion (26), so that early detection is essential. According to Vellone *et al.* (27), agenesis does not play any role in the process of canine eruption, while supernumeraries do.

One limitation of this study is that only patients under orthodontic treatment were evaluated. Therefore, some data should be interpreted with caution. For example, regarding the prevalence of rotational teeth, it may be possible that some patients had this condition already corrected at some point during orthodontic treatment.

## Conclusions

In summary, this study provides information about the dental anomalies observed in patients with NSCL/P, knowledge that is very important for craniofacial surgery planning. With an early diagnosis of these dental alterations and their orthodontic treatment, these abnormalities may be solved or minimized in cleft lip and palate patients and an aesthetic and functional pattern of dental arches can be obtained.
